# Bis(μ-5-carboxyl­ato-1-carboxyl­ato­methyl-2-oxidopyridinium)-κ^2^ *O* ^5^:*O* ^1^;κ^2^ *O* ^1^:*O* ^5^-[diaqua­(phenan­throline-κ^2^ *N*,*N*′)manganese(II)] dihydrate

**DOI:** 10.1107/S1600536809017668

**Published:** 2009-05-20

**Authors:** Mei-Xiang Jiang, Yun-Long Feng

**Affiliations:** a Zhejiang Key Laboratory for Reactive Chemistry on Solid Surfaces, Institute of Physical Chemistry, Zhejiang Normal University, Jinhua, Zhejiang 321004, People’s Republic of China

## Abstract

The  centrosymmetric binuclear title complex, [Mn_2_(C_8_H_5_NO_5_)_2_(C_12_H_8_N_2_)_2_(H_2_O)_4_]·2H_2_O, was obtained by the reaction of manganese chloride with 5-carb­oxy-1-carboxy­methyl-2-oxidopyridinium and 1,10-phenanthroline. The Mn^II^ atom is coordinated by two N atoms from the 1,10-phenanthroline ligand, two O atoms from two 5-carboxyl­ato-1-carboxyl­atomethyl-2-oxidopyridinium ligands and two water mol­ecules, leading to a distorted octahedral MnN_2_O_4_ environment. Inter­molecular O—H⋯O hydrogen bonds link neighbouring mol­ecules into a layer structure parallel to (001).

## Related literature

For the synthesis of compounds with multicarboxyl­ate ligands and metal centers, see: He *et al.* (2008 ▶); Huang *et al.* (2008 ▶); Jiang *et al.* (2009 ▶); Tong *et al.* (2005 ▶).
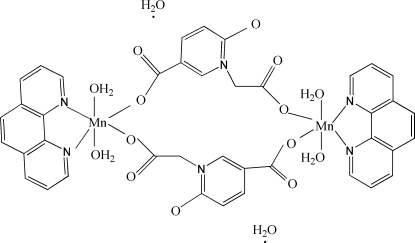


## Experimental

### 

#### Crystal data

[Mn_2_(C_8_H_5_NO_5_)_2_(C_12_H_8_N_2_)_2_(H_2_O)_4_]·2H_2_O
*M* *_r_* = 968.64
Triclinic, 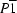

*a* = 7.7726 (11) Å
*b* = 9.9519 (14) Å
*c* = 15.411 (3) Å
α = 98.744 (10)°
β = 103.553 (10)°
γ = 110.252 (7)°
*V* = 1051.1 (3) Å^3^
*Z* = 1
Mo *K*α radiation
μ = 0.68 mm^−1^
*T* = 293 K
0.60 × 0.15 × 0.10 mm

#### Data collection

Bruker APEXII area-detector diffractometer
Absorption correction: multi-scan (*SADABS*; Sheldrick, 1996 ▶) *T*_min_ = 0.885, *T*_max_ = 0.934
19359 measured reflections
4779 independent reflections
3675 reflections with *I* > 2σ(*I*)
*R*_int_ = 0.171

#### Refinement

*R*[*F* ^2^ > 2σ(*F* ^2^)] = 0.051
*wR*(*F* ^2^) = 0.141
*S* = 1.00
4779 reflections
307 parameters
9 restraints
H atoms treated by a mixture of independent and constrained refinement
Δρ_max_ = 0.81 e Å^−3^
Δρ_min_ = −0.79 e Å^−3^

### 

Data collection: *APEX2* (Bruker, 2006 ▶); cell refinement: *SAINT* (Bruker, 2006 ▶); data reduction: *SAINT*; program(s) used to solve structure: *SHELXS97* (Sheldrick, 2008 ▶); program(s) used to refine structure: *SHELXL97* (Sheldrick, 2008 ▶); molecular graphics: *SHELXTL* (Sheldrick, 2008 ▶); software used to prepare material for publication: *SHELXTL*.

## Supplementary Material

Crystal structure: contains datablocks I, global. DOI: 10.1107/S1600536809017668/at2783sup1.cif

Structure factors: contains datablocks I. DOI: 10.1107/S1600536809017668/at2783Isup2.hkl

Additional supplementary materials:  crystallographic information; 3D view; checkCIF report

## Figures and Tables

**Table 1:** Hydrogen-bond geometry (Å, °)

*D*—H⋯*A*	*D*—H	H⋯*A*	*D*⋯*A*	*D*—H⋯*A*
O1*W*—H1*WA*⋯O4^i^	0.831 (16)	1.967 (18)	2.773 (2)	163 (3)
O1*W*—H1*WB*⋯O3*W*^ii^	0.822 (15)	1.920 (15)	2.730 (2)	169 (3)
O2*W*—H2*WA*⋯O4	0.807 (16)	1.998 (15)	2.783 (2)	164 (2)
O2*W*—H2*WA*⋯O5	0.807 (16)	2.57 (3)	2.984 (2)	114 (2)
O2*W*—H2*WB*⋯O1^iii^	0.856 (16)	1.959 (16)	2.806 (2)	170 (3)
O3*W*—H3*WA*⋯O1	0.833 (17)	1.962 (19)	2.775 (2)	165 (3)

Symmetry codes: (i) 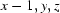; (ii) 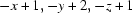; (iii) 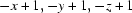.

**Fig. 1.**
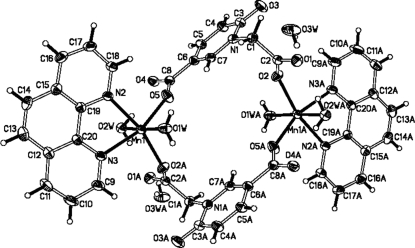
A view of the molecule of (I), showing the atom-labelling scheme. Displacement ellipsoids are shown at the 30% probability level [Symmetry code: (A) -x + 1, -y + 1, -z + 1].

**Fig. 2.**
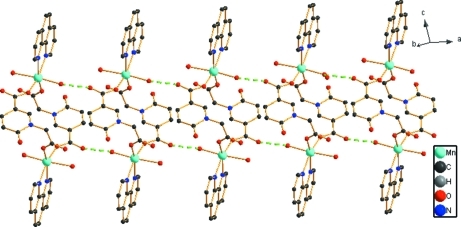
A view of the one-dimensional chain of the title compound. The O—H···O interactions are depicted by dashed lines.

## supplementary crystallographic information

### Comment

There is intensely research on the synthesis of compounds with multicarboxylate ligands and metal centers for their potential applications and coloful coordination methods. A large number of these compounds have been synthesized (He *et al.*, 2008; Huang *et al.*, 2008; Jiang *et al.*,2009; Tong *et al.*, 2005). As illustrated in Fig. 1, the Mn^II^ atom is coordinated by two nitrogen atoms from one 1,10-phenanthroline molecule, two oxygen atoms from two 5-carboxyl-1-carboxymethyl-2-oxidopyridinium ligands and two wate molecules. Four coordinated atoms of N2, N3, O5 and O2A constitute the base of the octahedral, whereas O1W and O2W atoms occupy the apical position. The intermolecular hydrogen bonds play an important role in the formation of the one-dimensional chain. As shown in Fig. 2. The intermolecular O—H···O hydrogen bonds link the neibouring molecules to a one-dimensional chain.

### Experimental

A mixture of 0.5 mmol 5-carboxyl-1-carboxymethyl-2-oxidopyridinium, 0.5 mmol 1,10-phenanthroline and 0.5 mmol of manganese chloride in 10 ml distilled water was stirred for 30 min at 323 K, then the reaction mixture was filtered and well shaped yellow crystals of the title compound was obtained from the mother liquor by slow evaporation at room temperature for several days.

### Refinement

The H atoms bonded to C atoms were positioned geometrically [aromatic C—H 0.93 Å and aliphatic C—H = 0.97 Å, *U*_iso_(H) = 1.2*U*_eq_(C)]. The H atoms bonded to O atoms were located in a difference Fourier maps and refined with O—H distance restraints of 0.85 and *U*_iso_(H) = 1.5*U*_eq_(O).

### Crystal data

**Table:**

[Mn_2_(C_8_H_5_NO_5_)_2_(C_12_H_8_N_2_)_2_(H_2_O)_4_]·2H_2_O	*V* = 1051.1 (3) Å^3^
*M**_r_* = 968.64	*Z* = 1
Triclinic, *P*1	*F*(000) = 498
Hall symbol: -P 1	*D*_x_ = 1.530 Mg m^−^^3^
*a* = 7.7726 (11) Å	Mo *K*α radiation, λ = 0.71073 Å
*b* = 9.9519 (14) Å	θ = 2.8–27.5°
*c* = 15.411 (3) Å	µ = 0.68 mm^−^^1^
α = 98.744 (10)°	*T* = 293 K
β = 103.553 (10)°	Block, yellow
γ = 110.252 (7)°	0.60 × 0.15 × 0.10 mm

### Data collection

**Table:**

Bruker APEXII area-detector diffractometer	4779 independent reflections
Radiation source: fine-focus sealed tube	3675 reflections with *I* > 2σ(*I*)
graphite	*R*_int_ = 0.171
ω scans	θ_max_ = 27.5°, θ_min_ = 2.8°
Absorption correction: multi-scan (*SADABS*; Sheldrick, 1996)	*h* = −10→10
*T*_min_ = 0.885, *T*_max_ = 0.934	*k* = −12→12
19359 measured reflections	*l* = −19→20

### Refinement

**Table:**

Refinement on *F*^2^	Primary atom site location: structure-invariant direct methods
Least-squares matrix: full	Secondary atom site location: difference Fourier map
*R*[*F*^2^ > 2σ(*F*^2^)] = 0.051	Hydrogen site location: inferred from neighbouring sites
*wR*(*F*^2^) = 0.141	H atoms treated by a mixture of independent and constrained refinement
*S* = 1.00	*w* = 1/[σ^2^(*F*_o_^2^) + (0.083*P*)^2^ + 0.0025*P*] where *P* = (*F*_o_^2^ + 2*F*_c_^2^)/3
4779 reflections	(Δ/σ)_max_ = 0.001
307 parameters	Δρ_max_ = 0.81 e Å^−^^3^
9 restraints	Δρ_min_ = −0.79 e Å^−^^3^

### Special details

**Table:**

Geometry. All e.s.d.'s (except the e.s.d. in the dihedral angle between two l.s. planes) are estimated using the full covariance matrix. The cell e.s.d.'s are taken into account individually in the estimation of e.s.d.'s in distances, angles and torsion angles; correlations between e.s.d.'s in cell parameters are only used when they are defined by crystal symmetry. An approximate (isotropic) treatment of cell e.s.d.'s is used for estimating e.s.d.'s involving l.s. planes.
Refinement. Refinement of *F*^2^ against ALL reflections. The weighted *R*-factor *wR* and goodness of fit *S* are based on *F*^2^, conventional *R*-factors *R* are based on *F*, with *F* set to zero for negative *F*^2^. The threshold expression of *F*^2^ > σ(*F*^2^) is used only for calculating *R*-factors(gt) *etc*. and is not relevant to the choice of reflections for refinement. *R*-factors based on *F*^2^ are statistically about twice as large as those based on *F*, and *R*- factors based on ALL data will be even larger.

### Fractional atomic coordinates and isotropic or equivalent isotropic displacement parameters (Å^2^)

**Table:**

	*x*	*y*	*z*	*U*_iso_*/*U*_eq_	
Mn1	0.34180 (4)	0.38197 (3)	0.24659 (2)	0.03192 (14)	
C1	0.8430 (3)	0.9583 (2)	0.60141 (17)	0.0399 (5)	
H1A	0.7365	0.9575	0.5533	0.048*	
H1B	0.9335	1.0605	0.6284	0.048*	
C2	0.7670 (3)	0.89610 (18)	0.67529 (14)	0.0334 (4)	
C3	1.1327 (3)	0.9121 (2)	0.60386 (16)	0.0416 (5)	
C4	1.2123 (3)	0.8185 (2)	0.56435 (17)	0.0447 (5)	
H4	1.3421	0.8388	0.5905	0.054*	
C5	1.1068 (3)	0.7009 (2)	0.49014 (16)	0.0397 (5)	
H5	1.1640	0.6423	0.4663	0.048*	
C6	0.9079 (3)	0.66801 (19)	0.44911 (15)	0.0350 (5)	
C7	0.8321 (3)	0.75625 (19)	0.48720 (15)	0.0358 (5)	
H7	0.7016	0.7344	0.4620	0.043*	
C8	0.7808 (3)	0.5421 (2)	0.36561 (15)	0.0363 (5)	
C9	−0.0500 (3)	0.1365 (2)	0.09487 (19)	0.0561 (7)	
H9	−0.0641	0.0926	0.1433	0.067*	
C10	−0.1881 (4)	0.0671 (3)	0.0077 (2)	0.0672 (9)	
H10	−0.2900	−0.0226	−0.0012	0.081*	
C11	−0.1736 (3)	0.1298 (2)	−0.06335 (19)	0.0565 (7)	
H11	−0.2654	0.0839	−0.1212	0.068*	
C12	−0.0184 (3)	0.2654 (2)	−0.04935 (16)	0.0413 (5)	
C13	0.0048 (3)	0.3422 (3)	−0.11964 (18)	0.0506 (6)	
H13	−0.0821	0.2997	−0.1789	0.061*	
C14	0.1498 (3)	0.4747 (3)	−0.10162 (17)	0.0495 (6)	
H14	0.1607	0.5238	−0.1481	0.059*	
C15	0.2884 (3)	0.5414 (2)	−0.01126 (16)	0.0405 (5)	
C16	0.4418 (4)	0.6812 (2)	0.01077 (19)	0.0509 (6)	
H16	0.4584	0.7336	−0.0338	0.061*	
C17	0.5659 (3)	0.7382 (2)	0.09913 (19)	0.0509 (6)	
H17	0.6660	0.8312	0.1156	0.061*	
C18	0.5416 (3)	0.6564 (2)	0.16410 (17)	0.0429 (5)	
H18	0.6289	0.6962	0.2234	0.051*	
C19	0.2732 (3)	0.46827 (18)	0.05867 (14)	0.0331 (4)	
C20	0.1150 (3)	0.32712 (18)	0.04029 (14)	0.0338 (4)	
O1W	0.2082 (2)	0.51934 (15)	0.30069 (13)	0.0469 (4)	
H1WA	0.107 (3)	0.494 (3)	0.3146 (19)	0.056*	
H1WB	0.262 (3)	0.6101 (18)	0.3185 (18)	0.056*	
O1	0.6879 (2)	0.96382 (15)	0.71523 (12)	0.0473 (4)	
O2W	0.5101 (2)	0.24567 (16)	0.20164 (12)	0.0439 (4)	
H2WA	0.612 (3)	0.297 (2)	0.2403 (16)	0.053*	
H2WB	0.459 (3)	0.177 (2)	0.2261 (16)	0.053*	
O2	0.7879 (2)	0.78107 (15)	0.68951 (12)	0.0440 (4)	
O3	1.2212 (2)	1.02222 (18)	0.67190 (14)	0.0640 (6)	
O3W	0.5827 (3)	1.18225 (16)	0.65623 (18)	0.0688 (6)	
H3WA	0.632 (4)	1.129 (3)	0.680 (2)	0.083*	
H3WB	0.471 (3)	1.149 (3)	0.659 (2)	0.083*	
O4	0.84928 (19)	0.46805 (15)	0.32338 (12)	0.0449 (4)	
O5	0.6043 (2)	0.52140 (18)	0.34392 (12)	0.0526 (5)	
N1	0.9381 (2)	0.87521 (16)	0.56051 (13)	0.0361 (4)	
N2	0.3998 (2)	0.52424 (16)	0.14566 (13)	0.0351 (4)	
N3	0.0999 (2)	0.26257 (16)	0.11052 (13)	0.0390 (4)	

### Atomic displacement parameters (Å^2^)

**Table:**

	*U*^11^	*U*^22^	*U*^33^	*U*^12^	*U*^13^	*U*^23^
Mn1	0.03112 (19)	0.02749 (17)	0.0329 (2)	0.00883 (12)	0.00698 (15)	0.00713 (12)
C1	0.0513 (11)	0.0308 (8)	0.0417 (13)	0.0170 (8)	0.0180 (10)	0.0135 (8)
C2	0.0322 (9)	0.0295 (8)	0.0345 (12)	0.0102 (7)	0.0072 (8)	0.0069 (7)
C3	0.0390 (10)	0.0365 (9)	0.0403 (13)	0.0071 (8)	0.0112 (9)	0.0057 (8)
C4	0.0306 (9)	0.0466 (10)	0.0455 (14)	0.0107 (8)	0.0024 (9)	0.0055 (9)
C5	0.0340 (9)	0.0377 (9)	0.0433 (13)	0.0132 (8)	0.0079 (9)	0.0079 (8)
C6	0.0318 (9)	0.0330 (8)	0.0348 (12)	0.0092 (7)	0.0055 (8)	0.0098 (8)
C7	0.0331 (9)	0.0334 (8)	0.0360 (12)	0.0088 (7)	0.0066 (8)	0.0118 (8)
C8	0.0336 (9)	0.0343 (8)	0.0361 (12)	0.0094 (7)	0.0079 (8)	0.0094 (8)
C9	0.0537 (13)	0.0374 (10)	0.0535 (17)	−0.0012 (9)	0.0010 (12)	0.0158 (10)
C10	0.0565 (14)	0.0399 (11)	0.066 (2)	−0.0090 (10)	−0.0065 (13)	0.0106 (12)
C11	0.0491 (12)	0.0441 (11)	0.0491 (17)	0.0073 (9)	−0.0067 (11)	−0.0029 (10)
C12	0.0399 (10)	0.0406 (9)	0.0383 (13)	0.0177 (8)	0.0041 (9)	0.0038 (8)
C13	0.0523 (12)	0.0593 (13)	0.0370 (14)	0.0270 (11)	0.0037 (10)	0.0076 (10)
C14	0.0587 (13)	0.0624 (13)	0.0383 (14)	0.0315 (11)	0.0165 (11)	0.0235 (11)
C15	0.0467 (11)	0.0407 (9)	0.0425 (13)	0.0223 (9)	0.0184 (10)	0.0151 (9)
C16	0.0622 (14)	0.0437 (11)	0.0559 (16)	0.0202 (10)	0.0270 (12)	0.0265 (10)
C17	0.0540 (13)	0.0328 (9)	0.0608 (18)	0.0071 (9)	0.0225 (12)	0.0149 (10)
C18	0.0422 (10)	0.0306 (8)	0.0455 (15)	0.0061 (8)	0.0113 (10)	0.0039 (8)
C19	0.0364 (9)	0.0291 (8)	0.0360 (12)	0.0156 (7)	0.0120 (8)	0.0068 (7)
C20	0.0350 (9)	0.0301 (8)	0.0347 (12)	0.0144 (7)	0.0076 (8)	0.0050 (7)
O1W	0.0401 (8)	0.0323 (6)	0.0712 (13)	0.0146 (6)	0.0243 (8)	0.0096 (7)
O1	0.0589 (9)	0.0417 (7)	0.0532 (11)	0.0275 (7)	0.0266 (8)	0.0131 (7)
O2W	0.0446 (8)	0.0401 (7)	0.0439 (11)	0.0140 (6)	0.0143 (7)	0.0084 (7)
O2	0.0562 (9)	0.0403 (7)	0.0529 (11)	0.0258 (6)	0.0295 (8)	0.0251 (7)
O3	0.0481 (9)	0.0496 (9)	0.0649 (14)	0.0037 (7)	0.0082 (8)	−0.0152 (8)
O3W	0.0591 (10)	0.0344 (7)	0.1180 (19)	0.0198 (7)	0.0337 (12)	0.0212 (9)
O4	0.0352 (7)	0.0413 (7)	0.0505 (11)	0.0110 (6)	0.0120 (7)	0.0020 (7)
O5	0.0323 (7)	0.0601 (9)	0.0504 (11)	0.0180 (7)	0.0006 (7)	−0.0080 (8)
N1	0.0384 (8)	0.0304 (7)	0.0386 (11)	0.0108 (6)	0.0135 (7)	0.0104 (7)
N2	0.0344 (8)	0.0282 (7)	0.0383 (11)	0.0085 (6)	0.0113 (7)	0.0058 (6)
N3	0.0376 (8)	0.0286 (7)	0.0411 (11)	0.0059 (6)	0.0063 (8)	0.0085 (7)

### Geometric parameters (Å, °)

**Table:**

Mn1—O5	2.0761 (14)	C10—C11	1.348 (4)
Mn1—O2^i^	2.1066 (15)	C10—H10	0.9300
Mn1—O1W	2.1643 (15)	C11—C12	1.409 (3)
Mn1—N2	2.2768 (18)	C11—H11	0.9300
Mn1—N3	2.2788 (17)	C12—C20	1.411 (3)
Mn1—O2W	2.3237 (17)	C12—C13	1.428 (3)
C1—N1	1.454 (3)	C13—C14	1.341 (3)
C1—C2	1.518 (3)	C13—H13	0.9300
C1—H1A	0.9700	C14—C15	1.436 (3)
C1—H1B	0.9700	C14—H14	0.9300
C2—O1	1.245 (2)	C15—C19	1.394 (3)
C2—O2	1.254 (2)	C15—C16	1.411 (3)
C3—O3	1.251 (2)	C16—C17	1.370 (4)
C3—N1	1.391 (3)	C16—H16	0.9300
C3—C4	1.423 (3)	C17—C18	1.390 (3)
C4—C5	1.356 (3)	C17—H17	0.9300
C4—H4	0.9300	C18—N2	1.327 (2)
C5—C6	1.423 (3)	C18—H18	0.9300
C5—H5	0.9300	C19—N2	1.361 (3)
C6—C7	1.352 (3)	C19—C20	1.444 (2)
C6—C8	1.503 (3)	C20—N3	1.348 (3)
C7—N1	1.356 (2)	O1W—H1WA	0.831 (16)
C7—H7	0.9300	O1W—H1WB	0.822 (15)
C8—O4	1.240 (3)	O2W—H2WA	0.807 (16)
C8—O5	1.267 (2)	O2W—H2WB	0.856 (16)
C9—N3	1.324 (2)	O2—Mn1^i^	2.1066 (15)
C9—C10	1.401 (3)	O3W—H3WA	0.833 (17)
C9—H9	0.9300	O3W—H3WB	0.833 (17)
			
O5—Mn1—O2^i^	105.32 (7)	C10—C11—C12	119.5 (2)
O5—Mn1—O1W	89.47 (6)	C10—C11—H11	120.2
O2^i^—Mn1—O1W	90.24 (6)	C12—C11—H11	120.2
O5—Mn1—N2	90.90 (7)	C11—C12—C20	116.6 (2)
O2^i^—Mn1—N2	163.71 (6)	C11—C12—C13	123.4 (2)
O1W—Mn1—N2	88.34 (7)	C20—C12—C13	120.00 (19)
O5—Mn1—N3	162.47 (8)	C14—C13—C12	121.1 (2)
O2^i^—Mn1—N3	91.35 (6)	C14—C13—H13	119.5
O1W—Mn1—N3	95.98 (7)	C12—C13—H13	119.5
N2—Mn1—N3	72.68 (6)	C13—C14—C15	120.6 (2)
O5—Mn1—O2W	85.20 (6)	C13—C14—H14	119.7
O2^i^—Mn1—O2W	89.67 (6)	C15—C14—H14	119.7
O1W—Mn1—O2W	174.44 (6)	C19—C15—C16	117.6 (2)
N2—Mn1—O2W	93.29 (6)	C19—C15—C14	120.02 (19)
N3—Mn1—O2W	89.58 (6)	C16—C15—C14	122.4 (2)
N1—C1—C2	112.73 (16)	C17—C16—C15	118.7 (2)
N1—C1—H1A	109.0	C17—C16—H16	120.7
C2—C1—H1A	109.0	C15—C16—H16	120.7
N1—C1—H1B	109.0	C16—C17—C18	119.7 (2)
C2—C1—H1B	109.0	C16—C17—H17	120.1
H1A—C1—H1B	107.8	C18—C17—H17	120.1
O1—C2—O2	126.3 (2)	N2—C18—C17	123.2 (2)
O1—C2—C1	116.48 (17)	N2—C18—H18	118.4
O2—C2—C1	117.18 (19)	C17—C18—H18	118.4
O3—C3—N1	118.6 (2)	N2—C19—C15	123.24 (17)
O3—C3—C4	126.2 (2)	N2—C19—C20	117.09 (18)
N1—C3—C4	115.20 (17)	C15—C19—C20	119.66 (18)
C5—C4—C3	122.79 (19)	N3—C20—C12	123.26 (18)
C5—C4—H4	118.6	N3—C20—C19	118.13 (17)
C3—C4—H4	118.6	C12—C20—C19	118.59 (19)
C4—C5—C6	119.4 (2)	Mn1—O1W—H1WA	128.8 (17)
C4—C5—H5	120.3	Mn1—O1W—H1WB	122.9 (17)
C6—C5—H5	120.3	H1WA—O1W—H1WB	108 (2)
C7—C6—C5	117.71 (18)	Mn1—O2W—H2WA	96 (2)
C7—C6—C8	119.05 (17)	Mn1—O2W—H2WB	93.0 (18)
C5—C6—C8	123.23 (19)	H2WA—O2W—H2WB	104 (2)
C6—C7—N1	122.87 (18)	C2—O2—Mn1^i^	134.18 (16)
C6—C7—H7	118.6	H3WA—O3W—H3WB	104 (2)
N1—C7—H7	118.6	C8—O5—Mn1	140.26 (15)
O4—C8—O5	124.86 (19)	C7—N1—C3	121.96 (18)
O4—C8—C6	120.83 (18)	C7—N1—C1	119.42 (17)
O5—C8—C6	114.31 (19)	C3—N1—C1	118.32 (17)
N3—C9—C10	122.1 (2)	C18—N2—C19	117.50 (19)
N3—C9—H9	118.9	C18—N2—Mn1	126.40 (15)
C10—C9—H9	118.9	C19—N2—Mn1	116.08 (12)
C11—C10—C9	120.2 (2)	C9—N3—C20	118.22 (18)
C11—C10—H10	119.9	C9—N3—Mn1	125.89 (16)
C9—C10—H10	119.9	C20—N3—Mn1	115.89 (12)
			
N1—C1—C2—O1	178.22 (17)	N2—Mn1—O5—C8	−84.9 (3)
N1—C1—C2—O2	−2.5 (3)	N3—Mn1—O5—C8	−64.8 (4)
O3—C3—C4—C5	−179.9 (3)	O2W—Mn1—O5—C8	8.3 (3)
N1—C3—C4—C5	−0.4 (3)	C6—C7—N1—C3	−2.2 (3)
C3—C4—C5—C6	0.0 (4)	C6—C7—N1—C1	−175.7 (2)
C4—C5—C6—C7	−0.6 (3)	O3—C3—N1—C7	−179.1 (2)
C4—C5—C6—C8	178.7 (2)	C4—C3—N1—C7	1.5 (3)
C5—C6—C7—N1	1.6 (3)	O3—C3—N1—C1	−5.4 (3)
C8—C6—C7—N1	−177.63 (18)	C4—C3—N1—C1	175.09 (19)
C7—C6—C8—O4	172.8 (2)	C2—C1—N1—C7	87.7 (2)
C5—C6—C8—O4	−6.4 (3)	C2—C1—N1—C3	−86.1 (2)
C7—C6—C8—O5	−6.9 (3)	C17—C18—N2—C19	−0.3 (3)
C5—C6—C8—O5	173.9 (2)	C17—C18—N2—Mn1	−178.77 (18)
N3—C9—C10—C11	−1.5 (5)	C15—C19—N2—C18	1.4 (3)
C9—C10—C11—C12	0.2 (5)	C20—C19—N2—C18	−177.59 (18)
C10—C11—C12—C20	0.3 (4)	C15—C19—N2—Mn1	−179.97 (16)
C10—C11—C12—C13	−177.8 (3)	C20—C19—N2—Mn1	1.1 (2)
C11—C12—C13—C14	176.6 (2)	O5—Mn1—N2—C18	−9.93 (18)
C20—C12—C13—C14	−1.5 (4)	O2^i^—Mn1—N2—C18	164.7 (2)
C12—C13—C14—C15	1.3 (4)	O1W—Mn1—N2—C18	79.51 (18)
C13—C14—C15—C19	0.2 (3)	N3—Mn1—N2—C18	176.30 (19)
C13—C14—C15—C16	−179.3 (2)	O2W—Mn1—N2—C18	−95.17 (18)
C19—C15—C16—C17	−0.7 (3)	O5—Mn1—N2—C19	171.54 (14)
C14—C15—C16—C17	178.9 (2)	O2^i^—Mn1—N2—C19	−13.9 (3)
C15—C16—C17—C18	1.7 (4)	O1W—Mn1—N2—C19	−99.02 (14)
C16—C17—C18—N2	−1.3 (4)	N3—Mn1—N2—C19	−2.23 (13)
C16—C15—C19—N2	−0.9 (3)	O2W—Mn1—N2—C19	86.30 (14)
C14—C15—C19—N2	179.6 (2)	C10—C9—N3—C20	2.0 (4)
C16—C15—C19—C20	178.02 (19)	C10—C9—N3—Mn1	−177.0 (2)
C14—C15—C19—C20	−1.5 (3)	C12—C20—N3—C9	−1.4 (3)
C11—C12—C20—N3	0.2 (3)	C19—C20—N3—C9	176.9 (2)
C13—C12—C20—N3	178.5 (2)	C12—C20—N3—Mn1	177.73 (16)
C11—C12—C20—C19	−178.11 (19)	C19—C20—N3—Mn1	−3.9 (2)
C13—C12—C20—C19	0.1 (3)	O5—Mn1—N3—C9	161.2 (2)
N2—C19—C20—N3	1.9 (3)	O2^i^—Mn1—N3—C9	−0.9 (2)
C15—C19—C20—N3	−177.08 (18)	O1W—Mn1—N3—C9	−91.3 (2)
N2—C19—C20—C12	−179.65 (18)	N2—Mn1—N3—C9	−177.7 (2)
C15—C19—C20—C12	1.4 (3)	O2W—Mn1—N3—C9	88.7 (2)
O1—C2—O2—Mn1^i^	17.7 (3)	O5—Mn1—N3—C20	−17.9 (3)
C1—C2—O2—Mn1^i^	−161.47 (15)	O2^i^—Mn1—N3—C20	179.98 (15)
O4—C8—O5—Mn1	1.0 (4)	O1W—Mn1—N3—C20	89.60 (15)
C6—C8—O5—Mn1	−179.30 (18)	N2—Mn1—N3—C20	3.23 (14)
O2^i^—Mn1—O5—C8	96.6 (3)	O2W—Mn1—N3—C20	−90.35 (15)
O1W—Mn1—O5—C8	−173.3 (3)		

Symmetry codes: (i) −*x*+1, −*y*+1, −*z*+1.

### Hydrogen-bond geometry (Å, °)

**Table:**

*D*—H···*A*	*D*—H	H···*A*	*D*···*A*	*D*—H···*A*
O1W—H1WA···O4^ii^	0.83 (2)	1.97 (2)	2.773 (2)	163 (3)
O1W—H1WB···O3W^iii^	0.82 (2)	1.92 (2)	2.730 (2)	169 (3)
O2W—H2WA···O4	0.81 (2)	2.00 (2)	2.783 (2)	164 (2)
O2W—H2WA···O5	0.81 (2)	2.57 (3)	2.984 (2)	114 (2)
O2W—H2WB···O1^i^	0.86 (2)	1.96 (2)	2.806 (2)	170 (3)
O3W—H3WA···O1	0.83 (2)	1.96 (2)	2.775 (2)	165 (3)

Symmetry codes: (ii) *x*−1, *y*, *z*; (iii) −*x*+1, −*y*+2, −*z*+1; (i) −*x*+1, −*y*+1, −*z*+1.
